# South Indian Isolates of the *Fusarium solani* Species Complex From Clinical and Environmental Samples: Identification, Antifungal Susceptibilities, and Virulence

**DOI:** 10.3389/fmicb.2018.01052

**Published:** 2018-05-23

**Authors:** Mónika Homa, László Galgóczy, Palanisamy Manikandan, Venkatapathy Narendran, Rita Sinka, Árpád Csernetics, Csaba Vágvölgyi, László Kredics, Tamás Papp

**Affiliations:** ^1^MTA-SZTE “Lendület” Fungal Pathogenicity Mechanisms Research Group, Szeged, Hungary; ^2^Department of Microbiology, Faculty of Science and Informatics, University of Szeged, Szeged, Hungary; ^3^Division of Molecular Biology, Biocenter, Medical University of Innsbruck, Innsbruck, Austria; ^4^Aravind Eye Hospital and Postgraduate Institute of Ophthalmology, Coimbatore, India; ^5^Department of Medical Laboratory Sciences, College of Applied Medical Sciences, Majmaah University, Majmaah, Saudi Arabia; ^6^Greenlink Analytical and Research Laboratory India Private Limited, Coimbatore, India; ^7^Department of Genetics, Faculty of Science and Informatics, University of Szeged, Szeged, Hungary

**Keywords:** keratomycosis, *Fusarium solani* species complex, *F. falciforme*, molecular identification, antifungal susceptibility, *Drosophila melanogaster*, virulence

## Abstract

Members of the *Fusarium solani* species complex (FSSC) are the most frequently isolated fusaria from soil. Moreover, this complex solely affects more than 100 plant genera, and is also one of the major opportunistic human pathogenic filamentous fungi, being responsible for approximately two-third of fusariosis cases. Mycotic keratitis due to *Fusarium* species is among the leading causes of visual impairment and blindness in South India, but its management is still challenging due to the poor susceptibility of the isolates to conventional antifungal drugs. Aims of the present study were to isolate South Indian clinical and environmental FSSC strains and identify them to species level, to determine the actual trends in their susceptibilities to antifungal therapeutic drugs and to compare the virulence of clinical and environmental FSSC members. Based on the partial sequences of the translation elongation factor 1α gene, the majority of the isolates—both from keratomycosis and environment—were confirmed as *F. falciforme*, followed by *F. keratoplasticum* and *F. solani sensu stricto*. *In vitro* antifungal susceptibilities to commonly used azole, allylamine and polyene antifungals were determined by the CLSI M38-A2 broth microdilution method. The first generation triazoles, fluconazole and itraconazole proved to be ineffective against all isolates tested. This phenomenon has already been described before, as fusaria are intrinsically resistant to them. However, our results indicated that despite the intensive agricultural use of azole compounds, fusaria have not developed resistance against the imidazole class of antifungals. In order to compare the virulence of different FSSC species from clinical and environmental sources, a *Drosophila melanogaster* model was used. MyD88 mutant flies having impaired immune responses were highly susceptible to all the examined fusaria. In wild-type flies, one *F. falciforme* and two *F. keratoplasticum* strains also reduced the survival significantly. Pathogenicity seemed to be independent from the origin of the isolates.

## Introduction

The genus *Fusarium* is a large group of hyaline filamentous fungi firstly described by Link (1809). According to the recent literature, it comprises approximately 200–300 species belonging to 20–22 species complexes (O'Donnell et al., 2013, 2015; Al-Hatmi et al., 2016). Fusaria are common soil saprophytes; however, they are also known as phytopathogens (Coleman, 2016). Two *Fusarium* species were recently included in the list of the top ten plant pathogenic fungi with both economic and scientific importance (Dean et al., 2012). The members of this genus may also interact with plants as endophytic root colonizers (Bacon and Yates, 2006); furthermore, they may be responsible for a wide range of human infections in either immunocompetent or immunocompromised patients (Garnica and Nucci, 2013). In accordance with the current species complex descriptions, at least ten of them have been reported to have human pathogenic representatives (Al-Hatmi et al., 2016). Last but not least, plumbing systems are also proven environmental reservoirs of human-pathogenic *Fusarium* species (Short et al., 2011).

Taxonomy of the genus *Fusarium* is changing intensely since 2011, when the era of the dual nomenclature ended (Hawksworth et al., 2011) and a comprehensive phylogenetic study of the genus discovered that the traditionally known *Fusarium* is not monophyletic (Gräfenhan et al., 2011). Based on these results, Gräfenhan et al. (2011) proposed to restrict the name *Fusarium* to the *Gibberella* clade and at the same time to reallocate the medically important *Fusarium solani* species complex (FSSC) and *Fusarium dimerum* species complex (FDSC) to other genera. After the release of this study, Lombard et al. (2015) were the first who suggested to use *Neocosmospora solani* instead of *F. solani*, and *Neocosmospora falciformis* instead of *F. falciforme*. However, in this study we would like to follow a previously published proposal of Geiser et al. (2013) by keeping the historical concept of *Fusarium* and use the names well-known in medical mycology.

In South India, a frequent scenario of fungal keratitis (keratomycosis) is that agricultural workers are infected after a corneal injury caused by plant or soil materials during their regular activities (Dóczi et al., 2004; Homa et al., 2013). Based on the recent reports, *Fusarium* species—and among them the members of the FSSC—are the most frequently isolated causative agents of fungal keratitis in this region (Chakrabarti and Singh, 2011; Homa et al., 2013; Hassan et al., 2016). The FSSC comprises at least 60 haplotypes, out of which 22 have been reported to have clinical associations (van Diepeningen et al., 2014; Al-Hatmi et al., 2016) with poor susceptibility to commonly used antifungal drugs (Azor et al., 2007). As consequence of the narrow range of therapeutic options, the treatment of *Fusarium* keratitis is extremely challenging and the lack of a prompt and effective therapy often results in corneal opacification or complete blindness (Shukla et al., 2008). Therefore, the rapid identification of the causative agent and the determination of its antifungal susceptibility are essential to choose the best therapeutic option. Presumably, the intensive agricultural and clinical (mis)use of antifungal compounds have also influenced the current susceptibility profile of the genus (Al-Hatmi et al., 2016). Thus, besides clinical studies, it is also crucial to evaluate the development of antifungal resistance among environmental strains to follow up the impact of fungicides used in the field.

Among FSSC species, *F. falciforme* was the most prevalent species isolated from human mycotic keratitis in South India (Homa et al., 2013; Hassan et al., 2016; Tupaki-Sreepurna et al., 2017a,b). However, it is unclear what lies in the background of its dominance: its environmental frequency or its high virulence. FSSC is reported to be more virulent than other species complexes of the genus (Mayayo et al., 1999); however, the virulence of different FSSC species has not been compared before. To answer the questions above, virulence studies are inevitable.

The objectives of the present study were (I) to isolate FSSC strains from keratomycosis patients, agricultural source and natural environments in South India; (II) to identify the strains at the species-level using molecular methods; (III) to determine their *in vitro* susceptibilities to commonly used antifungal agents; (IV) to compare the species diversity and the antifungal susceptibility profiles of the clinical and environmental isolates; (V) to compare the virulence of different clinical and environmental FSSC members; and (VI) to present and discuss the clinical details of the investigated keratomycosis cases.

## Materials and methods

### Patients specimens and *Fusarium* isolates

A total of 22 *Fusarium* isolates derived from patients with keratomycoses attending the Aravind Eye Hospital and Postgraduate Institute of Ophthalmology (Coimbatore, Tamilnadu, India) along with 20 environmental FSSC isolates from the same region were investigated (Table 1). Corneal scrapings were performed by an ophthalmologist under strict aseptic conditions, on each base of the corneal ulcer using a Kimura's spatula after instillation of 4% preservative-free lidocaine. Materials obtained from scraping the leading edge and the base of the ulcers were inoculated directly onto 5% sheep blood agar, chocolate agar, potato dextrose agar (PDA) and into brain heart infusion broth without gentamicin sulfate (Himedia Laboratories, India). Sheep blood agar and chocolate agar plates were incubated at 37°C, while PDA plates and brain heart infusion bottles were incubated at 27°C for 3 weeks.

**Table 1:** Molecular identification and antifungal susceptibilities of *Fusarium* strains isolated from keratitis cases and environmental samples in South India.

**SZMC No**.	**Year of isolation**	**Origin**	**State**	**Species based on *TEF1***	**Accession No. of *TEF1***	**MIC (μg/ml)**							
						**AMB**	**CLT**	**ECN**	**FLC**	**ITC**	**KTC**	**NTM**	**TRB**
**CLINICAL ISOLATES**													
SZMC 11438	2005	Gobichettypalayam	Tamilnadu	*F. falciforme*	HE647902	2	8	2	>64	>32	32	16	64
SZMC 11439	2005	Erode	Tamilnadu	*F. falciforme*	HE647903	4	16	2	>64	>32	16	8	64
SZMC 11441	2005	Gobichettypalayam	Tamilnadu	*F. falciforme*	HE647906	8	16	2	>64	>32	32	8	4
SZMC 11407	2005	Coimbatore	Tamilnadu	*F. falciforme*	HE647909	0.25	8	1	>64	>32	8	8	4
SZMC 11408	2005	Palladam	Tamilnadu	*F. falciforme*	HE647910	0.5	8	1	>64	>32	16	8	16
SZMC 11442	2005	Coimbatore	Tamilnadu	*F. falciforme*	HE647911	4	16	2	>64	>32	16	8	32
SZMC 11443	2004	Palladam	Tamilnadu	*F. falciforme*	HE647914	4	16	1	>64	>32	8	8	16
SZMC 11411	2005	Kangeyam	Tamilnadu	*F. falciforme*	HE647915	4	8	2	>64	>32	32	8	32
SZMC 11412	2005	Kangeyam	Tamilnadu	*F. falciforme*	HE647916	2	16	2	>64	>32	32	8	32
SZMC 11414	2004	Palghat	Kerala	*F. keratoplasticum*	HE647919	2	8	1	>64	>32	8	8	16
SZMC 11447	2005	Harur	Tamilnadu	*F. falciforme*	HE647924	1	4	1	>64	>32	32	8	0.5
SZMC 11448	2005	Coimbatore	Tamilnadu	*F. falciforme*	HE647927	4	8	4	>64	>32	32	8	32
SZMC 11449	2004	Palghat	Kerala	*F. falciforme*	HE647928	8	16	2	>64	>32	16	16	32
SZMC 11419	2005	Coimbatore	Tamilnadu	*F. falciforme*	HE647929	4	8	8	>64	>32	64	8	32
SZMC 11425	2005	Krishnagiri	Tamilnadu	*F. falciforme*	HE647937	4	16	1	>64	>32	16	8	16
SZMC 11454	2005	Tirupur	Tamilnadu	*F. falciforme*	HE647944	4	2	1	>64	>32	8	8	0.5
SZMC 11455	2005	Tiruchengodu	Tamilnadu	*F. falciforme*	HE647945	4	8	4	>64	>32	64	16	16
SZMC 11456	2005	Kangeyam	Tamilnadu	*F. falciforme*	HE647946	0.5	8	0.5	32	>32	2	8	4
SZMC 11457	2005	Tirupur	Tamilnadu	*F. falciforme*	HE647948	4	8	4	>64	>32	32	8	64
SZMC 11431	2005	Erode	Tamilnadu	*F. falciforme*	HE647949	4	8	4	>64	>32	64	8	32
SZMC 11432	2005	Tirupur	Tamilnadu	*F. falciforme*	HE647950	1	8	2	>64	>32	8	8	16
SZMC 11458	2005	Ottanchatram	Tamilnadu	*F. falciforme*	HE647951	2	4	8	>64	>32	8	4	4
MIC range (μg/ml)						0.25–8	2–16	0.5–8	32–>64	>32	2–64	4–16	0.5–64
MIC_50_ (μg/ml)						4	8	2	>64	>32	16	8	32
GM MIC (μg/ml)						3.2	8.8	2.1	>64	>32	18.1	8.5	19.0
**ENVIRONMENTAL ISOLATES**													
SZMC 21329	2012	Soil, flower garden	Tamilnadu	*F. falciforme*	MG272421	4	16	8	>64	>32	16	16	64
SZMC 21330	2012	Soil, flower garden	Tamilnadu	*F. keratoplasticum*	MG272422	4	16	8	>64	>32	64	8	32
SZMC 21331	2012	Soil, outdoor flowerpot	Tamilnadu	*F. falciforme*	MG272423	2	16	2	>64	>32	64	8	4
SZMC 21332	2012	Soil, outdoor flowerpot	Tamilnadu	*F. falciforme*	MG272424	4	8	8	>64	>32	64	16	32
SZMC 21333	2012	Soil, outdoor flowerpot	Tamilnadu	*F. falciforme*	MG272425	4	8	8	>64	>32	16	16	32
SZMC 21334	2012	Soil, outdoor flowerpot	Tamilnadu	*F. falciforme*	MG272426	4	8	8	>64	>32	64	8	64
SZMC 21335	2012	Soil, banana tree	Tamilnadu	*F. falciforme*	MG272427	4	16	4	>64	>32	32	8	32
SZMC 21336	2012	Soil, banana tree	Tamilnadu	*F. falciforme*	MG272428	4	16	4	>64	>32	32	8	32
SZMC 21337	2012	Soil, banana tree	Tamilnadu	*F. falciforme*	MG272429	2	16	2	>64	>32	64	8	4
SZMC 21338	2012	Soil, garden	Tamilnadu	*F. falciforme*	MG272430	1	8	16	>64	>32	64	8	16
SZMC 21339	2012	Soil, yard	Tamilnadu	*F. falciforme*	MG272431	4	8	4	>64	>32	64	16	32
SZMC 21340	2012	Soil, yard	Tamilnadu	*F. falciforme*	MG272432	4	8	4	>64	>32	64	16	32
SZMC 21342	2012	Soil, yard	Tamilnadu	*F. falciforme*	MG272433	4	8	1	>64	>32	16	8	8
SZMC 21343	2012	Soil, park	Tamilnadu	*F. falciforme*	MG272434	4	8	2	>64	>32	16	8	16
SZMC 21344	2012	Soil, park	Tamilnadu	*F. falciforme*	MG272435	2	16	8	>64	>32	32	8	16
SZMC 21345	2012	Soil, maize field	Tamilnadu	*F. falciforme*	MG272436	4	8	2	>64	>32	8	8	16
SZMC 21346	2012	Soil, sorghum field	Tamilnadu	*F. falciforme*	MG272437	2	8	2	>64	>32	16	8	8
SZMC 21348	2012	Soil, unknown cultivated field	Tamilnadu	*F. solani s. str*.	MG272438	2	8	16	>64	>32	8	8	4
SZMC 21350	2012	Sorghum, root	Tamilnadu	*F. falciforme*	MG272439	2	8	2	>64	>32	4	8	16
SZMC 21351	2012	Tomato, root	Tamilnadu	*F. falciforme*	MG272440	4	16	1	>64	>32	2	8	16
MIC range (μg/ml)						1–4	8–16	1–16	>64	>32	2–64	8–16	4–64
MIC_50_ (μg/ml)						4	8	4	>64	>32	32	8	16
GM MIC (μg/ml)						3.0	10.6	4.0	>64	>32	24.3	9.5	17.8

*AMB, amphotericin B; CLT, clotrimazole; ECN, econazole; FLC, fluconazole; GM, geometric mean; ITC, itraconazole; KTC, ketoconazole; MIC, minimum inhibitory concentration; MIC_50_, MIC for 50% of the tested isolates; NTM, natamycin; SZMC, Szeged Microbiological Collection, University of Szeged, Szeged, Hungary; TEF1, translation elongation factor 1α; TRB, terbinafine*.

To isolate fusaria from environmental sources, soil and plant parts (i.e., root and stem) were collected from gardens, parks, yards and agricultural fields in the surrounding regions of Coimbatore in November 2012. One gram of each collected soil sample was suspended in 10 ml sterile distilled water. The stock solutions were diluted 10 and 100 times and spread over Rose Bengal-Chloramphenicol agar (Himedia Laboratories, India) plates. The collected plant parts were pre-washed in sterile distilled water, surface-sterilized in 75% ethanol for 5 min and in 95% ethanol for 5 min, then rinsed in sterile distilled water for three times to remove ethanol residues. The sterilized parts were cut into small pieces, placed on Rose Bengal-Chloramphenicol agar plates and incubated at 25°C for 72 h. All fungal colonies from Rose Bengal-Chloramphenicol agar were subcultured into PDA plates using the cross-streak method. Then *Fusarium*-like colonies were purified and identified by macro- and microscopic characteristics. From both clinical and environmental samples, the purified fungal colonies were sub-cultured and stored on PDA plates at 4°C until further investigations.

### Molecular identification

Isolates suspected to be *Fusarium* sp. based on their macromorphological characteristics and microscopic features were further subjected to molecular identification. All isolates were grown in Potato Dextrose Broth (Sigma-Aldrich, USA) at 25°C in a shaker (New Brunswick Scientific Co., Inc., USA) at 220 rpm for 5 days, and subsequently genomic DNA was extracted with the MasterPure Yeast DNA Purification Kit (Epicentre Biotechnologies, USA) in accordance with manufacturer's instructions. FSSC isolates were selected using the FSSC-specific PCR as described by He et al. (2011) and confirmed with an *Eco*RI digestion-based PCR-RFLP method (Homa et al., 2013). For the species-level identification of FSSC-positive isolates, the 5′ portions of translation elongation factor 1α (*TEF1*) coding region and introns were amplified (O'Donnell et al., 1998). After Sanger sequencing (LGC Genomics GmbH, Germany) the *TEF1* sequences were deposited in the GenBank (https://www.ncbi.nlm.nih.gov/nucleotide/) under the accession numbers listed in Table 1 and used as BLAST (Altschul et al., 1990) queries against the *Fusarium* MLST database (http://www.westerdijkinstitute.nl/fusarium/) (O'Donnell et al., 2010).

All the confirmed isolates were deposited in the Szeged Microbiological Collection (SZMC; http://szmc.hu/; http://www.wfcc.info/ccinfo/collection/by_id/987) under the strain numbers listed in Table 1.

### Phylogenetic analysis

Besides the FSSC strains isolated from clinical and environmental sources, two clinical members of the *F. dimerum* species complex (SZMC 11496 and SZMC 11540) were involved in the analysis as an outgroup. The sequences were aligned by Muscle v3.8.31 (Edgar, 2004) and manually refined in BioEdit v7.1.3.0 (Hall, 1999). Substitution models for the final alignment were selected by the AIC_c_ function in jModelTest 2.1.10 (Posada, 2008). Trees were inferred by Maximum Likelihood (ML) and Bayesian MCMC approaches. ML bootstrapping was performed in PhyML 3.0 under the TrN+G model of sequence evolution, using the nearest-neighbor interchange branch swapping algorithm and 1000 replicates of non-parametric bootstrap analysis (Guindon and Gascuel, 2003). ML bootstrap values >69% were considered as significant support (Soltis and Soltis, 2003). Bayesian MCMC analyses were run in MrBayes 3.1.2 (Huelsenbeck and Ronquist, 2001). One cold and three incrementally heated chains were run in two replicates sampling every 100th generation. Chain length was set to 10,000,000 generations and a burn-in value of 100 000 generations was chosen using the Tracer 1.4 software (Rambaut and Drummond, 2007). Post-burn-in trees were summarized in a 50% majority rule consensus tree in MrBayes. Posterior probabilities >0.94 were considered as significant.

### Antifungal susceptibility tests

Antifungal susceptibility tests were performed as described in the Clinical and Laboratory Standards Institute (CLSI) M38-A2 broth microdilution method (Clinical Laboratory Standards Institute, 2008). Pharma grade powders of amphotericin B (AMB), clotrimazole (CLT), econazole (ECN), fluconazole (FLC), itraconazole (ITC), ketoconazole (KTC), terbinafine (TRB) (Sigma-Aldrich, USA), and commercially available natamycin (NTM) eye drops (Lalitha et al., 2008a) (Natamet, 5% suspension, Sun Pharmaceutical Ind. Ltd., India) were included in the tests. Conidial suspensions were prepared in 0.85% saline solution from 5-day-old cultures grown on PDA plates and diluted in RPMI-1640 medium (Sigma-Aldrich, USA) adjusting the final inoculum density to 10^4^ CFU/ml. Fungal growth was evaluated after incubation for 48 h at 35°C without shaking. Minimal inhibitory concentration (MIC) was determined as the lowest concentration of an antifungal agent that inhibited completely the growth of the tested isolates compared to the drug-free control medium. For FLC and KTC, the MICs were defined as the lowest concentrations of the drugs that cause approximately 50% reduction in growth. MIC_50_ was determined as the MIC inhibiting the growth of 50% of all the tested isolates. *Aspergillus flavus* ATCC 204304 and *Candida krusei* ATCC 6258 were included as quality control strains. Each experiment was performed in triplicates.

### Survival experiments in *Drosophila melanogaster*

To examine the background of *F. falciforme* dominance in South Indian human keratomycosis cases, the virulence of six FSSC isolates, i.e., *F. falciforme* SZMC 11407, SZMC 11408, and SZMC 21332, *F. keratoplasticum* SZMC 11414 and SZMC 21330, and *F. solani s. str*. SZMC 21348 was examined in *D. melanogaster*.

Conidial suspensions were prepared with sterile phosphate buffer saline (PBS; 137 mM NaCl, 2.7 mM KCl, 10 mM Na_2_HPO_4_, 2 mM KH_2_PO_4_, pH 7.4) from 5-day-old cultures grown on PDA plates at 35°C. The final inoculum densities were adjusted to 1 × 10^7^ conidia/ml with PBS.

**Figure 1:**
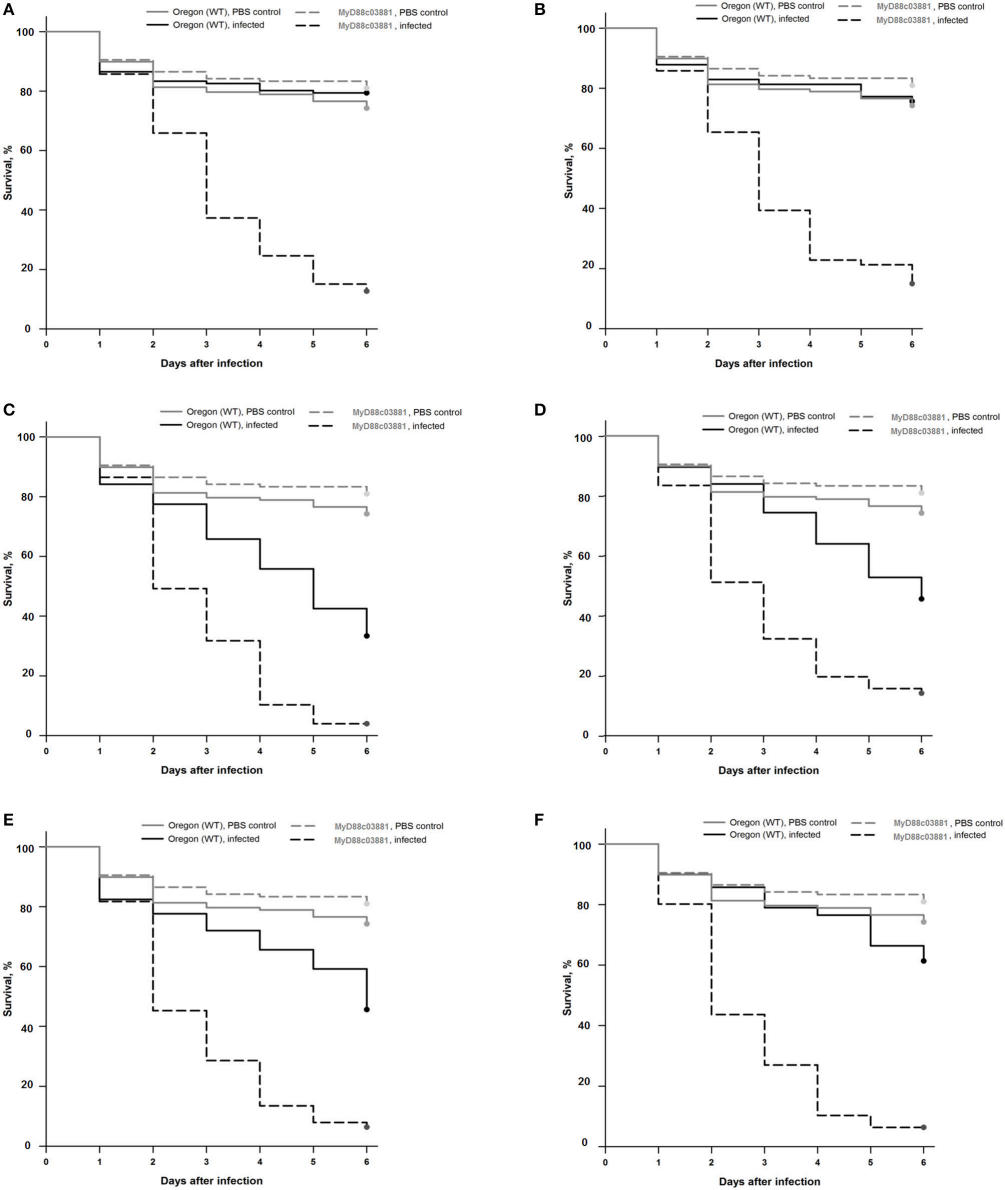
Survival rates of wild-type (Oregon) and MyD88c03881 mutant flies infected with clinical *F. falciforme* SZMC 11407 and SZMC 11408 **(A,B)**, clinical *F. keratoplasticum* SZMC 11414 **(C)**, environmental *F. keratoplasticum* SZMC 21330 **(D)**, environmental *F. falciforme* SZMC 21332 **(E)**, and environmental *F. solani s. str*. SZMC 21348 **(F)** strains. The control groups were injected with sterile PBS.

*Drosophila* stocks were raised and kept following the infection on standard cornmeal agar medium at 25°C. The Oregon R strain, originally obtained from the Bloomington stock center, was used as the wild type throughout the experiments. MyD88c03881 flies having impaired immune responses were described previously (Tauszig-Delamasure et al., 2002). Infection was performed by dipping a thin needle in a suspension of fungal conidia (10^7^ conidia/ml) or PBS for the uninfected control, and subsequently the thorax of the anesthetized fly was pricked. Flies were counted at different points of time to monitor survival. Flies were moved into fresh vials every other day. Each experiment was performed with approximately 60 flies for each genotype. The results shown in Figure 1 are representative of at least three independent experiments.

### Statistical analyses

All statistical analyses were performed in SigmaPlot (version 14.0). Two sample *t*-test was used to reveal significant differences between the antifungal susceptibility profiles of clinical and environmental isolates. Fisher's exact test was applied to compare the species composition of clinical and environmental isolates. Kaplan-Meier survival curves were generated in order to present the results of the survival experiments in *D. melanogaster*. The Log-Rank statistic was used to decide whether there is a statistically significant difference between the curves. To identify the group—or groups—of flies that differ from the others, the Holm-Sidak multiple comparison procedure was applied (Glantz, 2012). Significance level was set at *p* < 0.05.

### Ethics statement

Due to its observational nature, no formal ethics approval was required for this study. In order to protect the patients' anonymity, identifying information were not included in the manuscript.

## Results

### Clinical characteristics of patients with *fusarium* keratitis

Clinical data are available for all cases but one (Strain No. SZMC 11425) (Table 2). Out of the 22 keratitis isolates, 20 were isolated from patients residing in Tamilnadu, while the rest were from Kerala. Majority of the infections (*n* = 13) were registered between June and August. None of the patients had any underlying conditions. Half of the patients reported trauma as a predisposing factor, while 10 patients could not recall any injury prior to the infection. The severity of the ulcer was recorded as mild in six, moderate in seven and severe in eight cases. The most common therapeutic approach was the combined topical application of NTM, ITC, and ECN eye drops (*n* = 16), which were supplemented with systemic KTC in 12 cases. Surgical intervention (therapeutic penetrating keratoplasty, TPK) was needed in four severe cases, where the topical and systemic drugs could not improve the patients' condition. The registered final outcomes were as follows: 16 patients were healed completely; the therapy failed in five cases and one patient was lost to follow-up.

**Table 2:** Clinical details of *Fusarium* keratitis cases from South India.

**Strain No**.	**Month of presentation**	**History of trauma**	**VA at presentaion**	**Ulcer severity**	**Hypopyon**	**Antifungal treatment**		**Surgery**	**Subconjunctival injection**	**Perforation**	**FinalVA**	**Outcome**
						**Topical**	**Systemic**					
SZMC 11438	March	Mud	6/60	Moderate	No	NTM, ITC, ECN	No	No	No	No	6/12	Recovered
SZMC 11439	June	No	6/9	Moderate	No	NTM, ITC	No	No	No	No	6/9	Recovered
SZMC 11441	June	FB	HM	Severe	Yes	NTM, ITC, ECN	KTC	TKP	Yes	No	1/60	Failure
SZMC 11407	July	Dust	6/6	Moderate	No	NTM	No	No	No	No	6/6	Recovered
SZMC 11408	August	No	6/6	Mild	No	NTM, ITC, ECN	No	No	No	No	6/6	Recovered
SZMC 11442	July	Paddy husk	PL	Severe	Yes	NTM, ITC, ECN	KTC	TKP	Yes	No	1/60	Failure
SZMC 11443	December	Stick	1/60	Moderate	Yes	NTM, ITC, ECN	KTC	No	No	No	6/60	Recovered
SZMC 11411	July	No	6/12	Mild	CF	NTM, ITC, CLT	No	No	No	No	6/6	Recovered
SZMC 11412	July	No	6/60	Moderate	Yes	NTM, ITC, ECN	KTC	No	Yes	No	6/24	Recovered
SZMC 11414	December	No	FCF	Severe	Yes	NTM, ITC, ECN	KTC	TKP	No	No	6/24	Failure
SZMC 11447	March	No	2/60	Moderate	No	NTM, ITC, ECN	KTC	No	No	No	NA	Failure
SZMC 11448	June	Dust	6/24	Mild	No	NTM, ITC	No	No	No	No	6/24	Recovered
SZMC 11449	December	No	6/12	Mild	No	NTM, ITC, ECN	KTC	No	No	No	6/18	Recovered
SZMC 11419	August	FB	6/12	Moderate	No	NTM, ITC, ECN	No	No	No	No	6/24	Recovered
SZMC 11425	n.a.	n.a.	n.a.	n.a.	n.a.	n.a.	n.a.	n.a.	n.a.	n.a.	n.a.	n.a.
SZMC 11454	March	No	PL	Severe	Yes	NTM, ITC, ECN	KTC	No	No	No	PL	Recovered
SZMC 11455	March	No	1/60	Mild	No	NTM, ITC, ECN	No	No	No	No	6/9	Recovered
SZMC 11456	January	No	PL	Severe	Yes	NTM, ITC, ECN	KTC	TKP	No	Yes	NA	Failure
SZMC 11457	June	FB	6/9	Mild	No	NTM, ITC	No	No	No	No	6/6	Recovered
SZMC 11431	June	FB	PL	Severe	Yes	NTM, ITC, ECN	KTC	No	Yes	No	6/36	Recovered
SZMC 11432	June	Cow's tail	FCF	Severe	Yes	NTM, ITC	KTC	No	No	No	6/36	Recovered
SZMC 11458	June	Dust	3/60	Severe	Yes	NTM, ITC, ECN	KTC	No	Yes	No	6/24	Recovered

*ECN, econazole; FB, foreign body; FCF, counting fingers close to face; HM, hand motion; ITC, itraconazole; KTC, ketoconazole; n.a., not available; NTM, natamycin; PL, perceive light; SZMC, Szeged Microbiological Collection, University of Szeged, Szeged, Hungary; TKP, therapeutic keratoplasty; TN, Tamilnadu, VA, visual acuity*.

### Identification of the *fusarium* isolates based on molecular markers

**Figure 2:**
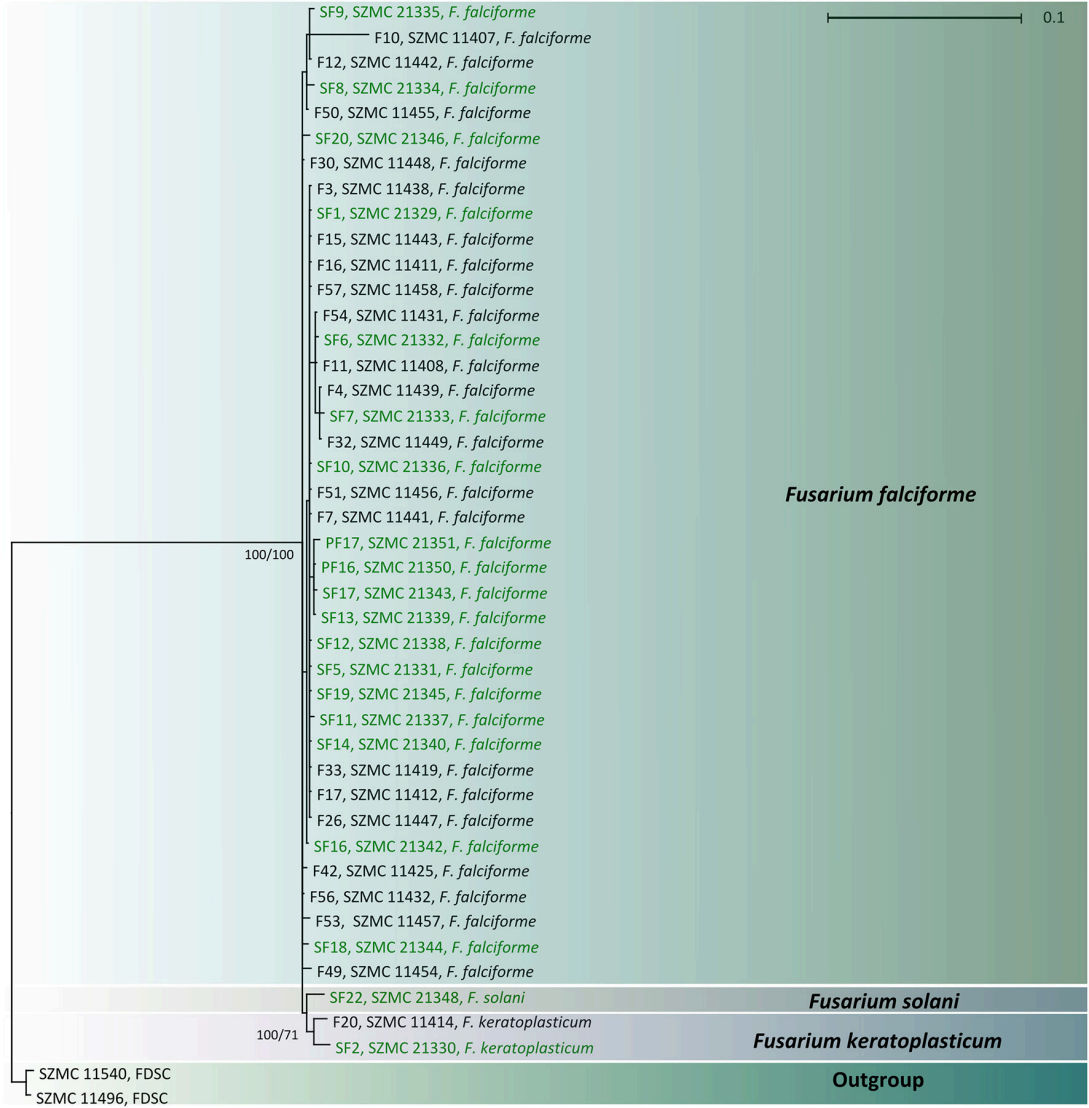
Combined phylogenetic tree based on the partial sequences of translation elongation factor 1α genes of clinical (indicated with black letters) and environmental (indicated with green letters) FSSC isolates. Only Bayesian posterior probability and ML bootstrap support values >0.95 and 60% are shown at the nodes.

BLAST searches with the partial *TEF1* sequences revealed that most of the isolates derived from both human keratomycoses and the environment belong to *F. falciforme* (*n* = 41; FSSC 3 + 4, O'Donnell et al., 2008). A clinical (SZMC 11414) and an environmental (SZMC 21330) isolate were identified as *F. keratoplasticum* (FSSC 2, Short et al., 2013), while another isolate from soil (SZMC 21348) was confirmed as *F. solani s. str*. (FSSC 5, Schroers et al., 2016; Table 1). Statistically significant association between the investigated FSSC species and their source was not detected. In order to examine the phylogenetic distribution of the isolates, a phylogenetic reconstruction was also performed using the above-mentioned *TEF1* locus, which confirmed the BLAST-based identifications (Figure 2).

### Antifungal susceptibilities

Table 1 summarizes the MIC values of the eight investigated antifungal agents. Clinical and environmental strains showed similar susceptibilities. However, environmental isolates proved to be significantly (*p* = 0.01) less susceptible to ECN than the clinical FSSC isolates. In all other cases, statistically significant differences were not detected between these two populations. The lowest MICs were recorded for AMB and ECN (0.25–16 μg/ml). MIC values of CLT and NTM were between 2 and 16 μg/ml, while the activities of TRB and KTC varied in the MIC ranges of 0.5–64 and 2–64 μg/ml, respectively. ITC and FLC proved to be ineffective in the tested concentration ranges.

### Virulence

In the case of wild type Oregon flies, the clinical *F. falciforme* strains SZMC 11407 and SZMC 11408 and the environmental FSSC 5 strain SZMC 21348 proved to be avirulent; the survival rates 6 days post infection (dpi) were 79 ± 21%, 76 ± 6%, and 61 ± 15% (Figures 1A,B,F). At the same time, infection with the *F. keratoplasticum* strains SZMC 11414 and SZMC 21330 and the environmental *F. falciforme* strain SZMC 21332 resulted in a significant reduction in the survival rate compared to the control group, the 6 dpi survival rates were 33 ± 18%, 46 ± 12%, and 46 ± 23% (Figures 1C–E), respectively. All six tested strains reduced the 6 dpi survival rates of the MyD88-mutant MyD88c03881 flies to 4–15% (Figures 1A–F).

## Discussion

The population of tropical/subtropical countries such as India is more prone to eye infections, especially to fungal keratitis caused by *Fusarium* spp. generally due to the climatic conditions. Regular monitoring of the disease is essential for its effective management (Lalitha et al., 2008b; Kredics et al., 2015).

As it is shown in Table 2, we found the highest incidence of FSSC keratitis cases in July. Previously, Lin et al. (2012) also observed an uneven distribution of *Fusarium* keratitis cases throughout the year in South India with a major peak of registered cases in July. This peak was associated with the windy season in June-July, when dust particles are presumed to be the main causes of ocular trauma (Lin et al., 2012). This theory is reinforced by the clinical records summarized in Table 2, where dust was mentioned as a predisposing factor for the infection only in June and July. A minor peak of keratitis cases in January—which was detected by Lin et al. (2012) and was attributed to the intensive agricultural activities of the harvest season resulting in elevated concentrations of conidia in the air and frequent ocular injuries due to soil or plant debris particles—was not observed in our study. Interestingly, Lin et al. (2012) also observed that environmental humidity (dry and wet season) was not a significant factor in the seasonal patterns of fungal keratitis.

We observed some major variations especially in the risk factors and treatment of *Fusarium* keratitis cases when compared our data with the study of Walther et al. (2017) from Germany. Based on the clinical details, trauma was the most frequently recorded predisposing factor for *Fusarium* keratitis in India (Bharathi et al., 2007; Tupaki-Sreepurna et al., 2017a), whereas keratomycoses were rare in temperate climates and more commonly associated with the use of soft contact lenses (Keay et al., 2011; Walther et al., 2017). As shown in Table 2, most of the patients at the Aravind Eye Hospital were treated with the topical applications of NTM, ECN and ITC along with systemic KTC, while in Germany, AMB and VRC were the most frequently used antifungals not just in topical, but also in invasive and systemic therapeutic approaches. Surgical intervention was performed in four out of the 21 cases in the present study, while Walther et al. (2017) reported that nine out of 15 cases required TPK. Despite the differing therapeutic approaches, comparing the outcomes we could not find any differences between the two investigations; therapeutic failures were reported 3/15 times (20.0%) by Walther et al. (2017) and 5/21 times (23.8%) in the present study.

Several papers reported that *Fusarium* species—particularly members of the FSSC—are the predominant etiological agents of keratomycosis (Table 3). However, based on the English language literature available in the PubMed (http://www.ncbi.nlm.nih.gov/pubmed) and Google Scholar (https://scholar.google.hu/) databases, species level identification is still not a common practice with *Fusarium* keratitis cases. In South India, *F. falciforme* proved to be the most common FSSC species followed by *F. keratoplasticum* (Homa et al., 2013; Hassan et al., 2016; Tupaki-Sreepurna et al., 2017a,b). These results were similar to those observed in the present study (Table 1). In the work of Tupaki-Sreepurna et al. (2017a), seven out of nine isolates were *F. keratoplasticum*, and the remaining two were identified as *F. falciforme*. This difference in the frequency of the two species could be explained by the small sample size. In contrast to these data from India, in Hong Kong and Singapore *F. keratoplasticum*, while in the USA, *F. petroliphilum, F. keratoplasticum* and *F. falciforme* were isolated from *Fusarium* keratitis cases. All of these species were identified in 2005 and 2006 during the multistate *Fusarium* keratitis outbreak associated with the use of Bausch and Lomb ReNu contact lens solution (Chang et al., 2006). Finally, in Germany *F. petroliphilum* and *F. keratoplasticum*, while in Turkey *F. solani s. str*. dominated among *Fusarium* keratitis isolates (Dalyan et al., 2015; Walther et al., 2017). *Fusarium lichenicola* was not isolated either from the environment or from clinical specimens in our study. According to our literature overview of *Fusarium* keratitis studies in Table 3, it is obvious that this species is an extremely rare causative agent of this disease. Previously, only Hassan et al. (2016) reported a single *F. lichenicola* isolate from keratomycosis from the Aravind Eye Hospital in Coimbatore, Tamilnadu.

**Table 3:** Literature overview of *Fusarium* keratitis studies with species-level data.

**Reference**	**Sampling period**	**Region**	**Species complex distribution (n)**	**Species distribution in the FSSC (n)**	**Gene(s) used for molecular analysis**	**Specific comments**
**ASIA**						
Sun et al., 2015	2002–2011	Central China	FSSC (386) FFSC (254) FOSC (11)	*F. solani s. str*. (132) *F. falciforme* (126)	*TEF1*	Species-level data are not available in English for the remaining 128 FSSC isolates
Homa et al., 2013	2010–2011	South India	FSSC (53) FDSC (6) FFSC (6) FOSC (3) FIESC (2)	*F. falciforme* (45) *F. keratoplasticum* (3) *F. solani s. str*. (1) FSSC 6 (1) FSSC 33 (2)	*TEF1*	–
Hassan et al., 2016	2012–2013	South India	FSSC (54) FDSC (7) FFSC (3) FOSC (1)	*F. falciforme* (45) *F. keratoplasticum* (8) *F. lichenicola* (1)	*TEF1, RPB2*	–
Tupaki-Sreepurna et al., 2017a	2012–2014	South India	FSSC (9) FSAMSC (1)	*F. keratoplasticum* (7) *F. falciforme* (2)	*TEF1, RPB2*	–
Tupaki-Sreepurna et al., 2017b	n.a.	South India	FSSC (43) FFSC (9) FDSC (1)	*F. falciforme* (36) *F. keratoplasticum* (6) Unnamed FSSC sp. (1)	*TEF1, RPB2, TUB2*, ITS	–
Chang et al., 2006	2005–2006	Hong Kong, Singapore	FSSC (20)	*F. keratoplasticum* (20)	*TEF1, RPB2*, ITS	Contact lens-associated cases
**EUROPE**						
Walther et al., 2017	2014–2015	Germany	FSSC (13) FOSC (6) FFSC (3)	*F. petroliphilum* (6) *F. keratoplasticum* (3) *F. falciforme* (1) *F. solani s. str*. (1) FSSC 9 (1) FSSC 25 (1)	*TEF1*	Mostly contact lens-associated cases
Dalyan et al., 2015	1995–2015	Turkey	FSSC (2) FFSC (1)	*F. solani s. str*. (2)	*TEF1*, ITS	
**NORTH-AMERICA**						
Chang et al., 2006	2005–2006	USA	FSSC (30) FOSC (7)	*F. petroliphilum* (13) *F. keratoplasticum* (12) *F. falciforme* (n.a.) FSSC 6 (n.a.) FSSC 7 (n.a.)	*TEF1, RPB2*, ITS	Contact lens-associated cases

*FDSC, Fusarium dimerum species complex; FFSC, Fusarium fujikuroi species complex; FIESC, Fusarium incarnatum-equiseti species complex; FOSC, Fusarium oxysporum species complex; FSAMSC, Fusarium sambucinum species complex; FSSC, Fusarium solani species complex; FSSC 6, FSSC 7, FSSC 9, FSSC 25 and FSSC 33, unnamed FSSC species; ITS, internal transcribed spacer region; n.a., not available; RPB2, the second largest subunit of RNA polymerase II gene; TEF1, translation elongation factor 1α; TUB2, beta-tubulin*.

Although our phylogeny (Figure 2) has been inferred using only the partial *TEF1* gene, it could be used to identify the isolates tested. *TEF1* is widely used to investigate the phylogenetic relationships of fusaria at the interspecific level (Debourgogne et al., 2012), but this locus alone is not appropriate to examine intraspecies relationships. According to O'Donnell et al. (2015), *TEF1* and the largest (*RPB1*) and the second largest subunit (*RPB2*) of the DNA-directed RNA polymerase II are the three most informative loci for phylogenetic species recognition in the genus *Fusarium*. Multilocus sequence typing (MLST) schemes including additional loci, e.g., the beta-tubulin gene, the internal transcribed spacer (ITS) region and the large ribosomal subunit gene (LSU) have also been proposed for fusaria (van Diepeningen et al., 2015). As it was previously described by Zhang et al. (2006) based on the phylogenetic analysis of the FSSC, we also found that clinical and environmental members of this species complex share a common evolutionary origin.

Susceptibility data on fusaria were not congruent in the literature (Table 4). According to Walther et al. (2017), FSSC and non-FSSC isolates may be easily separated based on their *in vitro* susceptibility to TRB. In their study, FSSC showed MICs higher than 32 μg/ml, while FOSC (*F. oxysporum* species complex) and FFSC (*F. fujikuroi* species complex) strains had MICs lower than 8 μg/ml (Walther et al., 2017). Our MIC data for the FSSC isolates did not confirm this suggestion; we observed a wide range of MICs for TRB (0.5–64 μg/ml). However, there are two fundamental differences between these reports: the geographical location and the species diversity. In contrast to the study of Walther et al. (2017), where *F. petroliphilum* and *F. keratoplasticum* were the most prevalent among the FSSC isolates, most of the strains in the present study were identified as *F. falciforme*.

**Table 4:** Literature overview of antifungal susceptibility data available for FSSC strains isolated from human keratomycoses.

**Reference**	**Sampling period**	**Tested isolates (n)**	**MIC ranges (μg/ml)**													
			**AMB**	**CLT**	CSP^*^	**ECN**	**FLC**	**ISV**	**ITC**	**KTC**	**MCN**	**NTM**	**NYS**	**PSC**	**TRB**	**VRC**
**INDIA**																
Homa et al., 2013	2010–2011	53	0.125–>64	4–>64	n.a.	8–>64	n.a.	n.a.	≥64	n.a.	n.a.	2–>64	n.a.	n.a.	1–>64	0.125–>64
Hassan et al., 2016	2012–2013	54	0.5–8	1–16	n.a.	2–8	8–16	n.a.	2–16	1–16	1–8	4–16	8–16	n.a.	n.a.	0.5–8
Tupaki-Sreepurna et al., 2017a	2012–2014	9	2–>32	n.a.	≥16	n.a.	n.a.	n.a.	16–>32	n.a.	n.a.	2-4	n.a.	n.a.	n.a.	8->32
**EUROPE**																
Walther et al., 2017	2014–2015	13	0.5–4	n.a.	16	n.a.	n.a.	16	16	n.a.	n.a.	4-16	n.a.	16	64	1-16
Dalyan et al., 2015	1995–2015	2	0.25–1	n.a.	>16	n.a.	>64	n.a.	>64	n.a.	n.a.	n.a.	n.a.	>16	n.a.	8-16

* *MECs, minimal effective concentration values*.

*AMB, amphotericin B; CLT, clotrimazole; CSP, caspofungin; ECN, econazole; FLC, fluconazole; ISV, isavuconazole; ITC, itraconazole; KTC, ketoconazole; MCN, micafungin; n.a., not available; NTM, natamycin; NYS, nystatin; PSC, posaconazole; TRB, terbinafine; VRC, voriconazole*.

In contrast to the currently reported susceptibility results, one of our previous studies revealed higher MIC values for all the tested antifungal drugs (Homa et al., 2013). However, in another South Indian keratitis study, Shobana et al. (2015) reported lower azole MICs (especially for ITC) for fusaria. According to Al-Hatmi et al. (2016), *Fusarium* spp. were intrinsically resistant to azoles. In agreement with this finding, we also found that ITC and FLC did not inhibit the growth of the investigated strains.

Triazole fungicides (i.e., hexaconazole, propiconazole, triadimefon, and tricyclazole) are commonly used for crop protection in India, especially in the Southern parts of the country (Chowdhary et al., 2012). The high exposure of environmental fungi to these compounds persisting in soil for a long time, may increase the risk of resistance development. For instance, the azole resistance of the common opportunistic human pathogen *Aspergillus fumigatus* was attributed to the non-medical use of these antifungals in the past few years (Van der Linden et al., 2011; Chowdhary et al., 2013; Azevedo et al., 2015; Berger et al., 2017). Although fusaria have not yet been investigated in this respect, we presume that resistance might emerge among them as a result of the permanent presence of fungicides in the environment.

When comparing our antifungal susceptibility data with previous studies, we also found similarities (Table 4). AMB and ECN were the most effective antifungal drugs against the majority of our isolates. Similarly, AMB showed the lowest MICs in other studies (Dalyan et al., 2015; Hassan et al., 2016; Walther et al., 2017). In accordance with the literature, all the MICs of NTM in our study were ≤16 μg/ml (Hassan et al., 2016; Tupaki-Sreepurna et al., 2017a; Walther et al., 2017), which is probably within the clinically achievable levels of this drug in eye tissue (Lalitha et al., 2008a).

Although species-specific clinical breakpoints are still not available for the genus *Fusarium*, CLSI epidemiological cutoff values (ECVs) were reported by Espinel-Ingroff et al. (2016) for AMB, posaconazole, voriconazole and ITC. These values may not able to help in predicting the clinical response to therapy but could possibly help to identify the so-called “non-wild-type isolates” or isolates that are less susceptible to the antifungal drugs. The authors defined “non-wild-type” as the population of strains in a species-drug combination with a detectable acquired resistance mechanism (Espinel-Ingroff et al., 2016). The ECVs for AMB and ITC are 8 μg/ml and 32 μg/ml, respectively. Based on these values, our isolates proved to be less susceptible to ITC (MIC > 32 μg/ml) than wild-type FSSC strains. At the same time, the MICs of AMB were below the ECV (Table 1).

*Drosophila melanogaster* has been previously described as a suitable invertebrate host model to study the *in vivo* virulence and pathogenesis of clinically important filamentous fungi (i.e., *Aspergillus* spp., Mucorales spp., *Scedosporium* spp. and *Fusarium* spp.) (Hamilos et al., 2012). This fly has a genetically tractable and well-characterized innate immune system, which is regulated by two distinct signaling pathways: the immune deficiency and the Toll pathways. While the first one is important in the defense against Gram-negative bacteria, the latter one has a key role in the immunity against Gram-positive bacteria and fungi (Lemaitre and Hoffmann, 2007). MyD88 is an adapter in the Toll pathway and its overexpression induces the expression of the antifungal peptide drosomycin (Tauszig-Delamasure et al., 2002).

Previously, Lamaris et al. (2007) infected wild-type Oregon and Toll-deficient flies with a clinical *F. verticillioides* (formerly *F. moniliforme*, a member of the FFSC) strain. Both wild-type and mutant flies were susceptible to this fungus, but in Toll-deficient flies a more acute infection and higher mortality rates were observed (Lamaris et al., 2007). As it was expected, we also found lower survival rates in case of the MyD88-mutants than the wild-type flies. Our results reconfirmed that MyD88 was essential for *D. melanogaster* to recognize and eliminate fusaria.

Although *D. melanogaster* proved to be highly susceptible to *F. keratoplasticum* (Figure 1) in our study, intraspecies differences in the virulence of *F. falciforme* isolates suggest that virulence is more like a strain-specific, than a species-specific feature. These results were in agreement with the hypothesis of Zhang et al. (2006), namely, that susceptible patients are infected with the most prevalent fungi in their environment.

In conclusion, our results confirmed that *F. falciforme* was the most prevalent species of the FSSC in South India isolated from both *Fusarium* keratitis patients and environmental sources. Antifungal susceptibility and virulence of clinical and environmental isolates were similar. However, we found major differences in the most common etiological agents, compared to North-American, European and other Asian countries. In the consequences of the high incidence of *Fusarium* keratitis and the significant rate of treatment failure, regular clinical studies are still necessary to develop an effective management of this disease in South India.

## Author contributions

TP: contributed to the design and implementation of the research, participated in drafting the manuscript; LK, VN, and CV: contributed to analyze the results and helped in drafting the manuscript; LG: participated in molecular identification and the antifungal susceptibility tests; PM: collected all the clinical data reported in the manuscript and participated in the morphological identification process; RS and ÁC: performed the virulence studies; MH: performed the antifungal susceptibility tests, drafted the manuscript and designed the figures and the tables. All authors read and approved the final manuscript.

### Conflict of interest statement

The authors declare that the research was conducted in the absence of any commercial or financial relationships that could be construed as a potential conflict of interest.
